# Pupillary responses to masked and gaze-averted faces

**DOI:** 10.3389/fpsyg.2025.1586186

**Published:** 2025-08-12

**Authors:** Cansu Malak, Funda Yildirim

**Affiliations:** ^1^Department of Brain and Cognitive Engineering, Korea University, Seoul, Republic of Korea; ^2^Department of Computer Engineering, Yeditepe University, Istanbul, Türkiye; ^3^Cognition, Data and Education Section, Faculty of Behavioural, Management and Social Sciences, University of Twente, Enschede, Netherlands

**Keywords:** emotion recognition, face mask, perceived gaze direction, facial expressions, eye-tracking, COVID-19

## Abstract

Face masks, a common practice during COVID-19, remain important in various cultural and medical contexts. Studies have shown how face masks affect our ability to recognize emotions, highlighting the role of facial features. Gaze direction plays a key role in modulating the identification of emotions, particularly in the presence of masks. So far, little is known about how gaze and masks influence emotion processing via physiological measures like pupil size. Here, we used pupillometry with 40 participants to investigate how emotion recognition (anger, fear, neutral) is affected by both gaze direction (direct, averted) and face mask conditions (mask, no mask). Behaviorally, our findings align with previous research, showing that the eye region plays a key role in identifying anger and neutral expressions more effectively than fear. Similarly, direct gaze improves accuracy for anger and neutral, while averted gaze enhances fear recognition. Pupillometry results revealed condition-specific changes in pupil size that partially mirrored the behavioral patterns, although no strong correlation with accuracy was found. However, pupil size was even more strongly modulated by recognition errors, with significantly greater dilation during incorrect trials across all emotions, especially for masked fearful faces, suggesting increased cognitive effort and ambiguity. The data also indicate compensatory processing mechanisms, when masks obscured parts of the face, participants appeared to rely more heavily on gaze direction and visible emotional cues. We propose that pupil dilation may reflect the cognitive load of emotion identification, providing important input for adaptive support applications in HCI and VR to improve user experiences.

## 1 Introduction

COVID-19 interventions have redefined social norms by introducing face masks, which cover around 60–70% of the lower face (Carbon, 2020) and hinder emotion recognition (Darwin, 1872; Nachson, 1995). While mask-wearing is common in certain cultures for religious (e.g., niqab or burqa) or environmental reasons, such as past epidemics (e.g., SARS; Coniam, 2005) or air pollution (e.g., in many Asian countries), Western societies faced unprecedented challenges adapting to masked communication after COVID-19 (Mheidly et al., 2020). Interpreting emotions and intentions in social interactions becomes even more challenging when considering gaze direction. To better understand how masked communication affects these cues, it is crucial to examine the role of gaze.

Regardless of culture, Ekman and Friesen (1975) revealed that people recognize six basic facial expressions (happiness, surprise, fear, sadness, anger, and disgust), each relying on specific facial cues (Beaudry et al., 2013). Eye-movement studies suggest people mostly focus on the mouth and eye regions (Eisenbarth and Alpers, 2011). The mouth region is especially important for detecting joy and disgust, whereas the eye region is more informative for fear and anger (Schurgin et al., 2014). A few other studies argue that the mouth region alone is as informative as the entire face in identifying certain expressions (Blais et al., 2012; Calvo and Nummenmaa, 2008).

Given our reliance on facial features, covering the lower face with a mask challenges emotion recognition by obscuring crucial emotional cues. Numerous studies show that masks hinder emotion recognition by limiting lower-face information (Parada-Fernández et al., 2022). Although masks reduce accuracy by 21–31% (Grundmann et al., 2021; Proverbio and Cerri, 2022), people still identify expressions relatively well, albeit more slowly (Williams et al., 2023). While people tend to focus on the eyes to compensate, few studies (e.g., Thomas et al., 2022) have examined their specific role in masked faces. Beyond the mouth and eye regions (i.e., eyes and brows), gaze direction is a key non-verbal cue for interpreting emotions, intentions, and attention (Lassalle and Itier, 2013). Therefore, examining the role of gaze direction is essential.

Gaze also serves as a mechanism for conveying one’s motivation to approach or avoid, thereby facilitating accurate interpretations of others’ intentions and enabling appropriate behavioral responses, such as fight or flight (Adams and Kleck, 2003; Sander et al., 2006). Direct gaze typically enhances recognition of approach-oriented emotions like anger or happiness, whereas averted gaze facilitates detection of avoidance-oriented emotions such as fear or sadness, as previously shown by Adams and Kleck (2003). While context can moderate these effects, perceived gaze direction remains a crucial factor in social communication, influencing how quickly and accurately we decode affective cues. In a recent study, Thomas et al. (2022) examined how wearing a face mask affected emotion recognition under different head orientations and gaze directions. Face masks amplified these effects, though recognition was unaffected by averted head orientation and only marginally influenced by gaze direction in the absence of a mask. However, to our knowledge, no research has specifically investigated how perceived gaze direction and face masks interact to influence pupillary responses during emotion recognition.

Pupillometry provides an objective index of cognitive and affective processes. Changes in pupil size reflect autonomic arousal and cognitive load (Beatty, 1982; Laeng et al., 2010). Threatening emotions like anger or fear often elicit greater pupil dilation (Bradley et al., 2008), and tasks with higher uncertainty or complexity also induce larger pupil sizes (Urai et al., 2017). However, pupil size may constrict when viewing highly attractive stimuli, reflecting aesthetic or self-monitoring responses (Liao et al., 2020). When the lower face is occluded by a mask, the added ambiguity may demand greater cognitive effort to discern emotions, potentially driving changes in pupil diameter. Because pupillary changes are largely involuntary, they are less susceptible to social desirability or conscious regulation (Kleberg et al., 2019; Partala and Surakka, 2003). This makes pupillometry an appealing measure for capturing subtle changes in mental effort or emotional arousal that might not emerge in reaction times or explicit recognition scores.

The interaction between gaze direction and emotional expression adds another layer of complexity to this process. Direct gaze often enhances the processing of approach-oriented emotions like anger, which elicit fast, intense reactions, whereas averted gaze benefits the recognition of avoidance-oriented emotions such as fear (Adams and Kleck, 2003). These differences in processing speed and accuracy may be reflected in distinct pupillary responses, providing a window into the underlying mechanisms of emotion recognition.

Accordingly, we predict that facial occlusion by a mask will significantly increase pupil dilation, particularly for emotions that rely heavily on lower facial features, reflecting heightened cognitive load when crucial cues are hidden. We also expect that mask presence and gaze direction will interact to shape pupillary responses differently for approach-versus avoidance-oriented emotions. For instance, pupil dilation might be stronger in situations where gaze and emotion send conflicting signals, such as when fear is paired with direct gaze, because the observer must resolve this ambiguity. Although we focus mainly on pupil dilation as a marker of emotional arousal and cognitive demand (Beatty, 1982; Laeng et al., 2010), it is important to acknowledge that pupil constriction may also occur under certain conditions (Hess and Polt, 1960). In particular, neutral or sad expressions, especially when masked—may be perceived as less emotionally intense or socially engaging, potentially leading to reduced sympathetic activation (Bradley et al., 2008; Van Steenbergen and Band, 2013). Moreover, constriction might reflect a low-arousal or disengaged state in response to ambiguous or non-threatening stimuli.

In this study, we employ behavioral and pupillometry measures to investigate how face masks and perceived gaze direction shape pupil dilation, reaction time, and emotion recognition accuracy. By doing so, we aim to provide a more comprehensive understanding of the cognitive and physiological processes involved in processing partially occluded facial expressions and their implications for real-world social interactions.

## 2 Method

### 2.1 Participants

We recruited forty-two volunteers (18–35 years old; 28 female; *M* = 24.57, *SD* = 4.19) with normal or corrected vision who participated in the study on a voluntary basis. The required sample size was determined *a priori* using G*Power (Faul et al., 2007) to achieve 80% power (1 − β = 0.80) at an α level of 0.05 for detecting a medium effect size (*f* = 0.25; Cohen, 1988) in a repeated-measures ANOVA with 12 within-subject conditions (3 emotions × 2 mask conditions × 2 gaze directions). This decision was further supported by similar studies investigating facial emotion recognition under mask conditions (e.g., Carbon, 2020). The analysis indicated that a minimum of 36 participants would be sufficient. We recruited 42 participants to account for potential data loss, and the final sample included 40 participants after applying exclusion criteria. Our study followed the WMA Declaration of Helsinki guidelines and was approved by the Yeditepe University Ethical Committee for Social Sciences.

### 2.2 Materials and Stimuli

**Figure 1:**
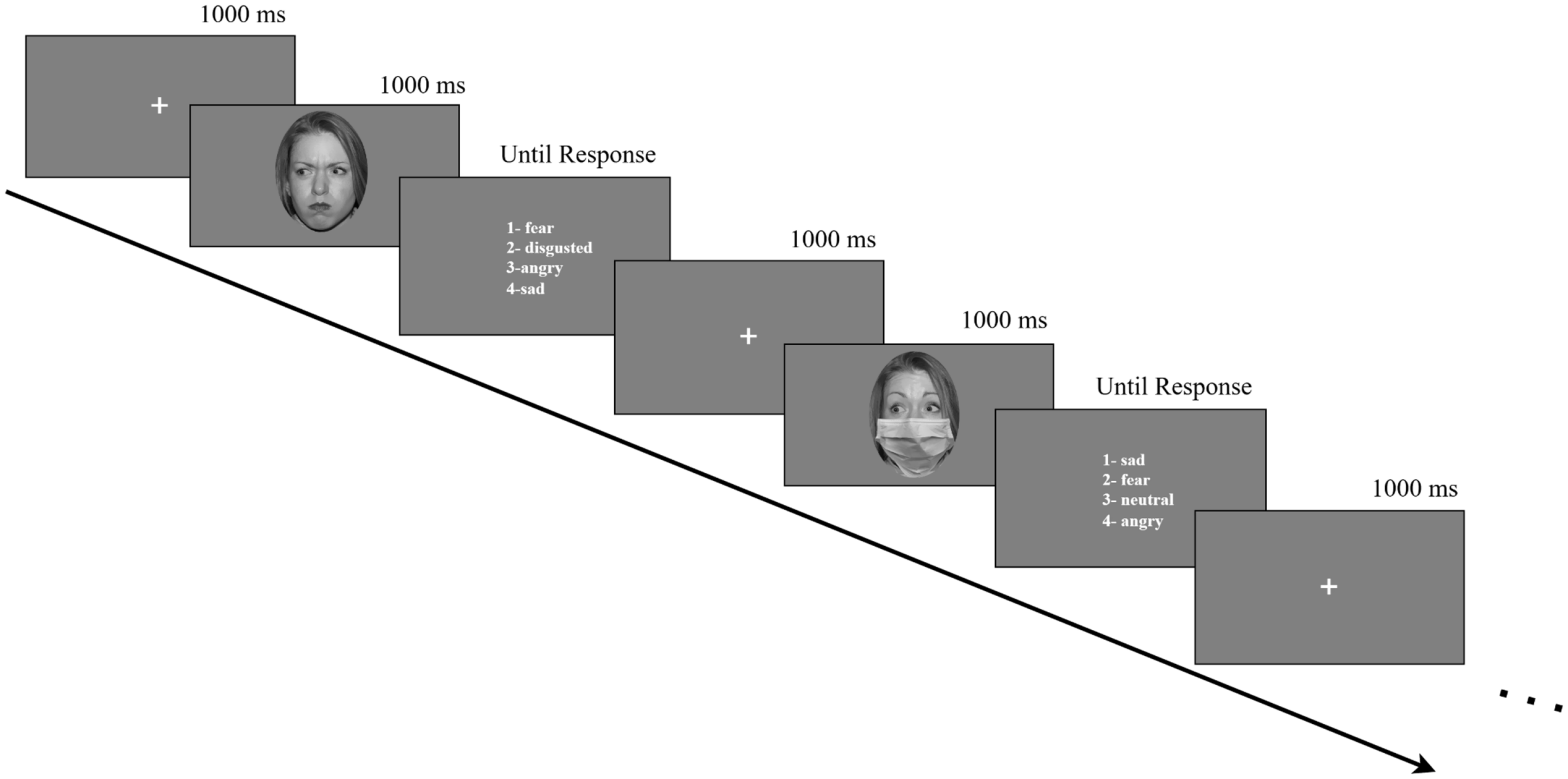
Example trial of the experiment. Facial images reproduced with permission from the NimStim Set of Facial Expressions (Tottenham et al., 2009), with use limited to model #1 as per dataset guidelines.

We used face images of four models (two female) from the NimStim database (Tottenham et al., 2009), depicting anger, fear, and neutral expressions. These specific expressions were selected based on their theoretical relevance: anger is an approach-oriented emotion that is more easily recognized with direct gaze, while fear is an avoidance-oriented emotion that tends to be recognized more accurately with averted gaze (Adams and Kleck, 2003). Neutral expressions were included as a baseline. We elliptically cropped the faces to eliminate hair and background cues and converted them to black-and-white. We then edited each image to display two gaze directions (direct/averted) and two mask conditions (mask/no mask) using Adobe Photoshop (Figure 1). To create averted gaze stimuli, we followed the method described by Lassalle and Itier (2013), manually repositioning the irises toward the outer corners of the eyes to simulate a leftward or rightward gaze. While we did not use a specific angular measurement, this manipulation visually created a clearly distinguishable averted gaze relative to the direct gaze condition. Each individual face model was presented in both direct and averted gaze versions. Averted gaze direction (left or right) was visually balanced across models and expressions. Afterward, we standardized luminance with SHINE (Willenbockel et al., 2010). We aligned the images to ensure consistency across facial areas. The face images were 1,608 × 2,048 pixels and displayed centrally on a gray background, subtending approximately 7.8° (width) × 10.2° (height) of visual angle at a 60 cm viewing distance. We programmed the task in MATLAB (R2021a; MATLAB, 2019) using Psychtoolbox-3 (Brainard, 1997; Pelli, 1997).

### 2.3 Procedure

We conducted the experiment in a dark room. Each participant sat 60 cm away from the computer screen and maintained this distance using a chin rest throughout the experiment. We presented stimuli on an ASUS VG278H 27” 3D monitor with a 16:9 aspect ratio, 1,920×1,080 resolution, and a 120 Hz refresh rate. Eye movements were recorded with a 60-Hz eye tracker and a 0.5-1-degree visual angle (Gazepoint GP3, Gazepoint Research Inc., Vancouver, Canada).

#### 2.3.1 Emotion Recognition Task

We conducted the experiment in two sessions, each consisting of 96 stimuli. All conditions—emotion (anger, fear, neutral), gaze direction (direct, averted), and face mask (mask, no mask)—were fully crossed and repeated eight times per session. Stimulus presentation was fully randomized for each participant, meaning that consecutive trials could feature faces with similar conditions. Each trial began with a central fixation cross, displayed for 1,000 ms, followed by a face stimulus presented for 1,000 ms. After the face disappeared, participants were shown four emotion labels randomly selected from a fixed set of six (angry, fearful, neutral, disgusted, sad, and confused). To avoid visual overload, maintain task efficiency, and reduce the likelihood of strategic responding, only four of the six predefined emotion labels were shown randomly on each trial, with the correct label always included. Participants responded by pressing keys 1–4 on the keyboard. The response screen remained visible until they answered, allowing them unlimited response time.

#### 2.3.2 Eye-Tracking Recordings and Data Preprocessing

We recorded eye movements throughout the emotion recognition task. Before each session, we conducted a five-point calibration and a four-point validation. To ensure data quality, trials with less than 75% valid pupil data within the stimulus presentation window (1,000 ms) were excluded from further analysis. Additionally, participants with less than 75% valid pupil data across all trials for either eye were excluded from the study, resulting in a final sample of 40 participants. For consistency, only right-eye data were used in all analyses. For preprocessing, we applied a low-pass filter using a third-order Butterworth filter with a 2 Hz cutoff frequency and then Z-scored each participant’s data for normalization. No interpolation was performed on the raw pupil data to account for blinks or other transient data loss; instead, these missing data points were treated as NaNs in the subsequent averaging process. Trials with more than 50% missing pupil data were excluded from the analysis. This approach ensured that only trials with sufficiently reliable pupil traces contributed to the mean pupil size computations per condition. On average, less than 10% of trials were excluded per participant due to insufficient pupil data. Z-score normalization was computed individually for each participant using the mean and standard deviation of their pupil signal across all valid trials. Therefore, “greater pupil size” refers to higher z-scored values relative to a participant’s own average pupil diameter, rather than absolute dilation from a pre-stimulus baseline.

### 2.4 Statistical Analyses

We conducted repeated-measures ANOVAs to analyze the effects of emotion (angry, fear, neutral), gaze direction (direct, averted), and face mask (mask, no mask) on recognition accuracy and reaction time. A 3 (Emotion) × 2 (Gaze Direction) × 2 (Mask) within-subjects design was used. Interaction effects were examined first, followed by *post hoc* comparisons using Bonferroni correction where appropriate. Effect sizes are reported using partial eta squared (ηp^2^). All reported accuracy and reaction time values reflect participant-level means, averaged across all valid trials in each condition. Accuracy values were computed per participant by averaging correct responses within each experimental condition, and then group-level means were derived by averaging across participants. To explore the physiological index of cognitive load, we also conducted a 3 (Emotion) × 2 (Mask) × 2 (Gaze Direction) repeated-measures ANOVA on pupil size (Z-score normalized). Additional paired-samples t-tests compared pupil responses between correct and incorrect recognition trials. To further investigate whether gaze allocation across facial regions may have confounded the observed pupil dilation effects, we conducted a supplementary 3 (Emotion) × 2 (Mask) × 2 (Gaze Direction) repeated-measures ANOVA on fixation counts to predefined Areas of Interest (AOIs: eyes and mouth). Full results are presented in Supplementary Tables S1, S2, and Supplementary Figure S1. All analyses were performed in JASP (Love et al., 2019), and statistical values are reported in accordance with APA guidelines. Study data are available on the Open-Source Framework.^1^

## 3 Results

### 3.1 Emotion Recognition Task

A 3 (Emotion) × 2 (Gaze Direction) × 2 (Face Mask) repeated-measures ANOVA revealed several significant interaction effects. Most notably, there was a significant three-way interaction between emotion, gaze direction, and face mask, *F*(2, 82) = 4.94, *p* = 0.009, *ηp^2^* = 0.11. This was further qualified by significant two-way interactions between emotion and face mask, *F*(2, 82) = 13.81, *p* < 0.001, *ηp^2^* = 0.25; emotion and gaze direction, *F*(1.57, 64.35) = 39.88, *p* < 0.001, *ηp^2^* = 0.49; and face mask and gaze direction, *F*(1, 41) = 10.54, *p* = 0.002, *ηp^2^* = 0.21. *Post hoc* comparisons revealed that recognition performance for fearful faces was significantly lower in the mask condition (*M* = 57.22, *SD* = 17.95) than in the no-mask condition (*M* = 65.77, *SD* = 19.30), *p* < 0.001. However, no significant mask-related differences were observed for angry or neutral expressions (*ps* > 0.05). Regarding gaze direction, neutral and angry expressions were recognized more accurately with direct gaze (neutral: *M* = 83.48, *SD* = 10.47; angry: *M* = 90.40, *SD* = 9.37) than with averted gaze (neutral: *M* = 62.05, *SD* = 21.95, *p* < 0.001; angry: *M* = 83.63, *SD* = 12.13, *p* = 0.005). Recognition performance for fearful expressions did not significantly differ based on gaze direction (*p* > 0.05). Additionally, direct gaze yielded higher recognition accuracy than averted gaze in both masked (*M* = 76.14, *SD* = 9.84 vs. *M* = 70.54, *SD* = 13.92, *p* < 0.001) and unmasked conditions (*M* = 79.76, *SD* = 10.68 vs. *M* = 68.60, *SD* = 13.85, *p* < 0.001). To further explore the overall impact of each variable, we also examined main effects. There was a significant main effect of emotion, *F*(2, 82) = 50.95, *p* < 0.001, *ηp^2^* = 0.55, with participants recognizing angry expressions most accurately (*M* = 87.02, *SD* = 10.08), followed by neutral (*M* = 72.77, *SD* = 14.54) and fearful expressions (*M* = 61.50, *SD* = 17.26), all *ps* < 0.001. A significant main effect of gaze direction was also observed, *F*(1, 41) = 52.51, *p* < 0.001, *ηp^2^* = 0.56, with higher overall accuracy for direct gaze (*M* = 77.95, *SD* = 9.38) than averted gaze (*M* = 69.57, *SD* = 12.95). The main effect of face mask on recognition performance was not significant (*p* > 0.05).

**Figure 2:**
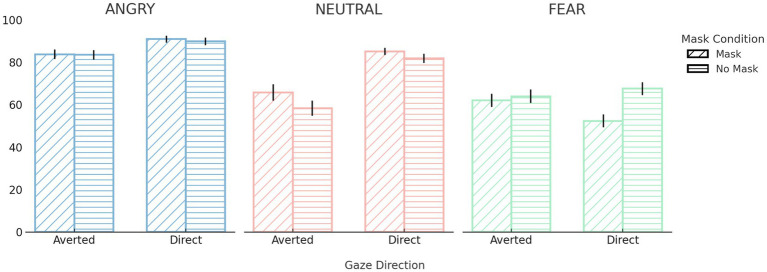
Performance during the emotion recognition task. Emotion recognition accuracy (%) across emotion, gaze direction, and face mask conditions and error bars represent standard errors of the mean (SE). For full descriptive statistics across all emotion × gaze × mask combinations, see Supplementary Table 3.

In summary, recognition accuracy was highest for angry expressions and lowest for fear, with direct gaze facilitating recognition especially for angry and neutral faces. While the presence of face masks significantly impaired recognition of fearful expressions, it had minimal impact on the recognition of angry and neutral faces (see Figure 2).

Additionally, we examined the reaction times of participants during the emotion recognition task using a repeated-measures ANOVA. In line with our analytical strategy, we first assessed interaction effects. Significant interaction effects were observed for emotion × mask, *F*(1.78, 1194.33) = 3.59, *p* = 0.03, *ηp^2^* = 0.005, and emotion × gaze direction, *F*(1.75, 1175.68) = 25.52, *p* < 0.001, *ηp^2^* = 0.04. However, the mask × gaze direction interaction, *F*(1, 671) = 0.15, *p* = 0.70, and the three-way interaction among emotion, mask, and gaze, *F*(2, 1124.95) = 1.16, *p* = 0.31, were not statistically significant. To further interpret these interactions, we conducted Bonferroni-adjusted pairwise comparisons. Participants responded significantly faster to fearful expressions in the unmasked condition (*M* = 2438.10 ms, *SD* = 2085.79) than in the masked condition (*M* = 2354.89 ms, *SD* = 1866.72), *p* = 0.012. No significant mask-related differences were found for angry or neutral expressions (*ps* > 0.05). Regarding gaze effects, angry expressions were recognized faster with direct gaze (*M* = 1805.63 ms, *SD* = 1376.58) than with averted gaze (*M* = 1926.62 ms, *SD* = 1452.85), *p* = 0.005. Similarly, neutral expressions also elicited significantly faster responses with direct gaze (*M* = 1705.51 ms, *SD* = 1679.67) than with averted gaze (*M* = 2680.74 ms, *SD* = 3127.88), *p* < 0.001. In contrast, no significant difference in reaction times was observed for fearful expressions between direct (*M* = 2174.57 ms, *SD* = 1842.58) and averted gaze (*M* = 2568.43 ms, *SD* = 2058.80), *p* = 0.24. In addition to these interactions, we observed significant main effects of emotion, *F*(1.89, 1269.35) = 52.49, *p* < 0.001, *ηp^2^* = 0.73, and gaze direction, *F*(1, 671) = 105.22, *p* < 0.001, *ηp^2^* = 0.14. However, the main effect of mask was not significant, *F*(1, 671) = 0.13, *p* = 0.71. Overall, participants responded fastest to angry faces (*M* = 1866.12 ms, *SD* = 1570.00), followed by neutral (*M* = 2117.63 ms, *SD* = 2620.63), and slowest to fearful expressions (*M* = 2396.50 ms, *SD* = 1963.29). Reaction times were also significantly shorter for direct gaze (*M* = 1911.90 ms, *SD* = 1728.22) than for averted gaze (*M* = 2341.60 ms, *SD* = 2409.38), *p* < 0.001.

Reaction time analyses complemented the accuracy results, showing that participants responded most quickly to angry faces, followed by neutral, and most slowly to fearful ones. Direct gaze consistently led to faster responses for angry and neutral expressions, but not for fearful faces, suggesting that the effect of gaze direction on processing speed depends on emotional content. These findings support the idea that anger is processed as an approach-oriented emotion, prompting rapid evaluation, while fear, especially under ambiguous conditions such as face masks, may require more cognitive effort and time to interpret accurately (see Supplementary Figure 3).

### 3.2 Pupillometry (Pupil Diameter)

Repeated-measures ANOVA on pupil size revealed several significant interaction effects. There was a significant interaction between emotion and mask, *F*(2, 1,276) = 6.84, *p* = 0.001, *ηp^2^* = 0.011, as well as an interaction between emotion and gaze direction, *F*(2, 1,276) = 3.23, *p* = 0.040, *ηp^2^* = 0.005. Additionally, a significant three-way interaction among emotion, gaze direction, and mask was observed, *F*(2, 1,276) = 4.57, *p* = 0.010, *ηp^2^* = 0.007. *Post hoc* comparisons indicated that pupil size was significantly smaller when viewing angry faces with direct gaze (*M* = −0.08, *SD* = 0.89) compared to fearful faces with averted gaze (*M* = 0.02, *SD* = 0.94), *p* = 0.029; fearful faces with direct gaze (*M* = 0.023, *SD* = 0.80), *p* = 0.016; and neutral faces with averted gaze (*M* = 0.039, *SD* = 1.02), *p* = 0.005. When faces were masked, fearful expressions elicited significantly greater pupil dilation (*M* = 0.06, *SD* = 0.85) compared to angry expressions (*M* = −0.052, *SD* = 1.08), *p* = 0.005. Angry faces with a mask and direct gaze evoked smaller pupil sizes (*M* = −0.12, *SD* = 0.97) than the same faces with averted gaze (*M* = 0.013, *SD* = 1.18), *p* = 0.026, as well as compared to fearful faces with a mask and averted gaze (*M* = 0.03, *SD* = 0.93), *p* = 0.024; neutral faces without a mask and averted gaze (*M* = 0.07, *SD* = 1.02), *p* < 0.001; and fearful faces with a mask and direct gaze (*M* = 0.09, *SD* = 0.75), *p* < 0.001. Furthermore, fearful faces with a mask and direct gaze led to greater pupil dilation (*M* = 0.09, *SD* = 0.75) compared to the same faces without a mask (*M* = −0.04, *SD* = 0.84), *p* = 0.016; and compared to neutral faces with a mask and direct gaze (*M* = 0.06, *SD* = 1.50), *p* = 0.014. Finally, a significant main effect of gaze direction was also observed, *F*(1, 638) = 8.95, *p* = 0.003, *ηp^2^* = 0.014, with averted gaze (*M* = 0.025, *SD* = 1.03) inducing greater pupil dilation than direct gaze (*M* = −0.029, *SD* = 0.97). No significant main effects were found for emotion or mask independently (*ps* > 0.05).

**Figure 3:**
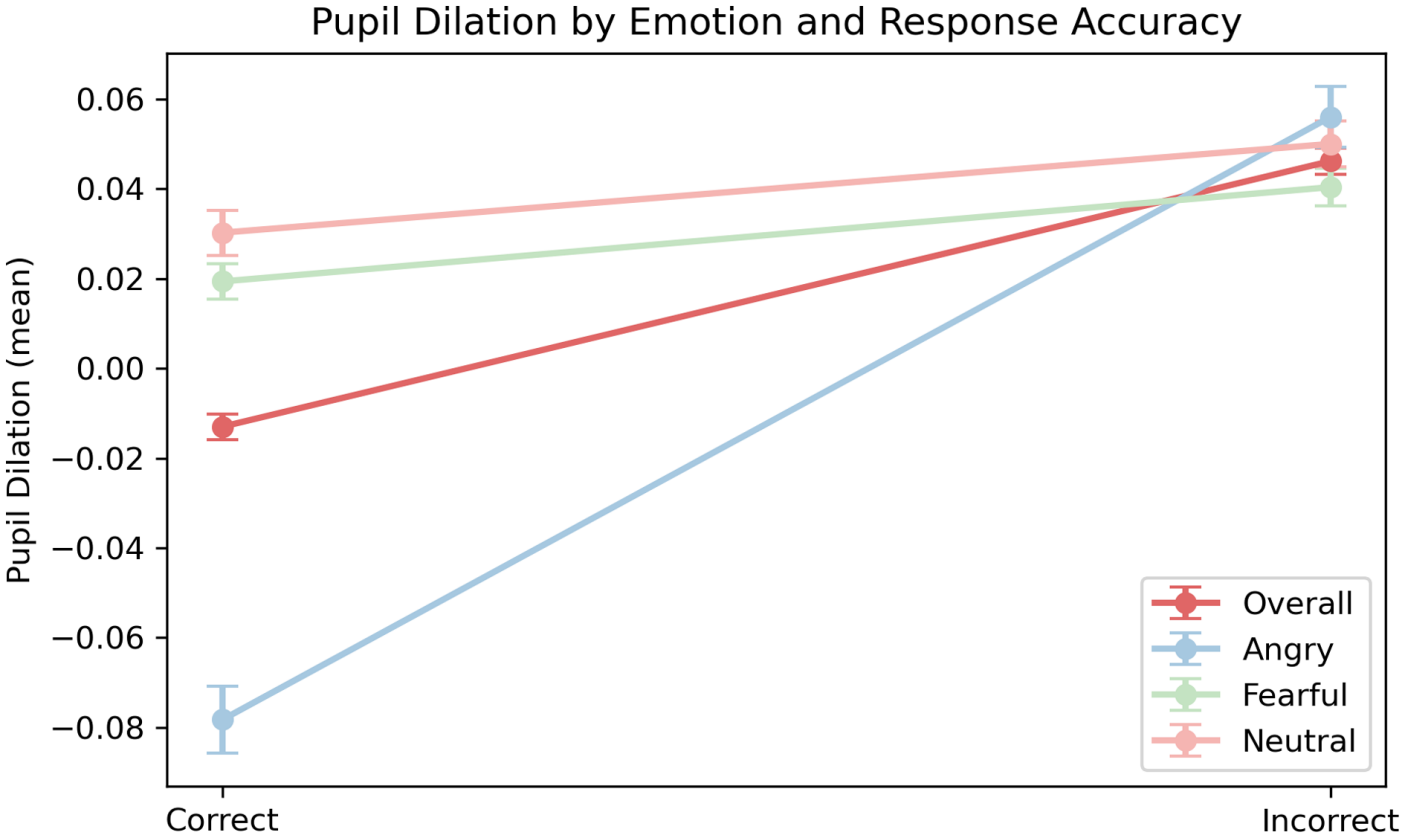
Pupil dilation by emotion and response accuracy. Mean pupil dilation (z-scored) is plotted as a function of response accuracy (correct vs. incorrect) across three emotion categories (angry, fearful, neutral), along with an overall average. Error bars represent standard errors of the mean (SE).

To examine whether pupil dilation reflects increased cognitive demands during emotion recognition, we conducted paired-samples *t*-tests comparing z-scored pupil size between correct and incorrect trials across emotional conditions. Overall, pupil dilation was significantly greater during incorrect trials than during correct trials, *t*(122,425) = −14.49, *p* < 0.001. When analyzed by emotion, this difference remained significant for all categories. For angry expressions, pupil size was significantly greater during incorrect trials, *t*(19,880) = −13.26, *p* < 0.001. For fearful and neutral expressions, pupil dilation was significantly greater during incorrect trials, *t*(61,097) = −3.66, *p* < 0.001, and *t*(41,446) = −2.73, *p* = 0.006, respectively. These results suggest that, even when individual baseline differences in pupil reactivity are accounted for through normalization, pupil responses remain modulated by recognition accuracy and emotional category. In all cases, incorrect recognition was associated with greater pupil dilation, potentially reflecting increased cognitive load or decision uncertainty under ambiguous or challenging conditions (see Figure 3).

**Figure 4:**
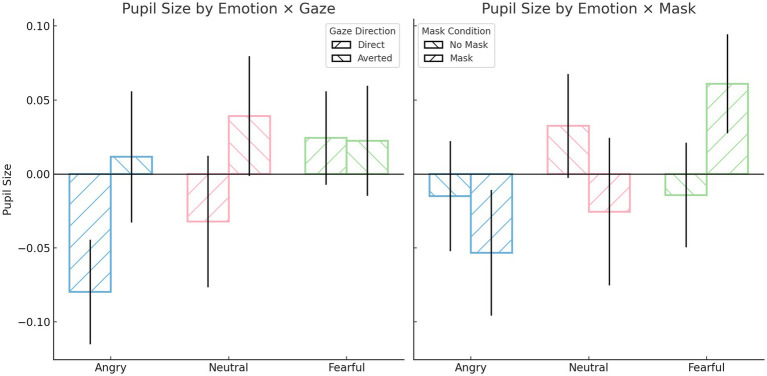
Participant’s pupil size during emotion recognition task. All values reflect participant-level means (*N* = 40), averaged across all valid trials per condition. Pupil size values are z-score normalized, with positive values indicating dilation above the participant’s average pupil size and negative values indicating constriction. Error bars represent standard errors of the mean (SE).

To summarize, pupil dilation was generally greater in response to fearful expressions, particularly when a face mask was present and gaze was direct, reflecting increased cognitive load under conditions of ambiguity. Additionally, averted gaze elicited greater pupil size overall compared to direct gaze, underscoring the role of gaze direction in modulating perceptual and cognitive effort during emotion recognition (see Figure 4).

Additionally, we conducted a Supplementary 3 (emotion) × 2 (mask) × 2 (gaze direction) repeated-measures ANOVA on fixation counts to predefined Areas of Interest (AOIs). For the eye region (AOI1), we found significant main effects of emotion and mask, as well as an emotion × mask interaction (all *ps* < 0.001). Similarly, for the mouth/mask region (AOI2), emotion and mask effects were significant, and again, a significant emotion × mask interaction emerged (all *ps* < 0.001). However, gaze direction and higher-order interactions (e.g., emotion × mask × direction) were not statistically significant in either AOI (*ps* > 0.05), suggesting that the observed pupil effects cannot be explained by differential fixation patterns. Detailed ANOVA results and fixation distributions are provided in Supplementary Tables S1, S2, and Supplementary Figure S1.

## 4 Discussion

In this study, we investigated how perceived gaze direction and face mask affect the recognition of anger, fear, and neutral expressions, as well as changes in pupil size. Our findings revealed three major insights: (1) face masks especially impaired the recognition of fear but had little or no negative impact on anger and for neutral expressions; (2) gaze direction affected recognition, benefiting anger and neutral faces with direct gaze while showing no significant difference for fear recognition based on gaze direction; and critically, (3) pupil dilation appeared to reflect cognitive load in ambiguous or incongruent conditions, significantly increasing during incorrect trials across all emotions and peaking under conditions of ambiguity, particularly for masked fearful expressions.

Our results showed that fear recognition performance dropped significantly with masks, suggesting participants relied heavily on lower-face information for fear. This corroborates with other studies stressing the importance of the mouth region for detecting fear (Calvo and Nummenmaa, 2008; Beaudry et al., 2013), and findings that face masks reduce fear recognition (Grahlow et al., 2022; Marini et al., 2021). In contrast, face masks did not affect anger or neutral recognition. Some studies suggest that the eye region alone is sufficient for identifying anger and fear (Calder et al., 2000), but others underscore the joint importance of mouth and eyes for certain emotions (Beaudry et al., 2013; Eisenbarth and Alpers, 2011). In line with Beaudry et al. (2013), our behavioral findings support the holistic processing of fear and underline the importance of the eye region for anger. Therefore, our findings on unmasked and masked faces show that eyes are sufficient for recognizing neutral and angry faces whereas fear recognition relies more on holistic or lower-face cues.

Further, our study underlines that gaze direction is important in emotion recognition. Gaze is a key nonverbal cue that conveys emotional states, attentional focus, and approach or avoidance tendencies (Adams and Kleck, 2003). Recognizing gaze direction is among the earliest developing visual skills (Farroni et al., 2002), and its communicative value becomes particularly salient when facial cues are limited, for instance, under face mask conditions. Direct gaze typically captures the observer’s attention and facilitates recognition of approach-oriented emotions like anger and neutral expressions (Adams and Kleck, 2003; Sander et al., 2006), while averted gaze has been linked to avoidance-oriented emotions such as fear (Böckler et al., 2014). Reflecting this pattern, while anger and neutral expressions were recognized more accurately with direct gaze, no significant gaze-related difference was observed for fear expressions, despite a slight numerical trend favoring averted gaze. Reaction time data further support this interpretation, showing that participants responded fastest to angry faces, followed by neutral, and slowest to fearful expressions. Direct gaze consistently led to faster responses for angry and neutral expressions, but crucially, not for fearful faces. This pattern of slowed responses for fear, especially under masked condition, directly mirrors the accuracy deficits and suggests that the processing of fear involves greater cognitive effort or ambiguity resolution, particularly when diagnostic cues are occluded. Also, RT results suggest that direct gaze facilitates not only accuracy but also processing speed, especially for anger.

To further explore how participants interpreted emotions beyond accuracy, we analyzed misclassification patterns across the six response options. As shown in Supplementary Figure S2, fearful expressions were most frequently confused with “confused” (31.6%) and “neutral” (14.2%) labels. Similarly, neutral expressions were occasionally misidentified as “confused” (11.8%) or “sad” (9.5%). These misclassifications, particularly fear of being labeled as “confused,” provide compelling behavioral evidence for the ambiguity participants experienced when decoding fearful expressions, especially under masked conditions. In contrast, angry expressions were rarely confused with other emotions, which highlights how clearly anger was recognized across different conditions.

Pupillometry results provided strong physiological evidence for the cognitive load associated with emotion recognition under ambiguity. Most significantly, pupil dilation was consistently greater during incorrect trials compared to correct trials across all emotional categories. This robust finding strongly supports the interpretation that pupil dilation reflects increased cognitive effort, decision uncertainty, or processing difficulty during the task, particularly when recognition fails. Fearful faces with a face mask also elicited greater pupil dilation. Here, greater pupil dilation reflects higher z-scored values relative to each participant’s own average pupil diameter, as described in the Methods section. Combined with our behavioral finding that participants identified fear more accurately in unmasked conditions, and its frequent misclassification as “confused,” these results suggest that pupil size reflects cognitive workload and decision-making processes during the emotion recognition task. Although we hypothesized that increased pupil dilation would be associated with higher recognition accuracy, our correlation analyses did not support a direct statistical link. Instead, the significant dilation on error trials indicates that pupil size primarily indexes the difficulty or ambiguity of the recognition process itself, rather than successful outcome. Pupil dilation appeared to reflect cognitive load in ambiguous or incongruent conditions, particularly for faces expressing fear with a face mask. *Post hoc* comparisons revealed that masked fearful faces with direct gaze evoked significantly greater pupil responses than the same emotion without a mask, as well as compared to masked angry and neutral faces under direct gaze (see Supplementary Figure S4). Furthermore, the overall main effect of gaze direction revealed greater pupil dilation for averted gaze compared to direct gaze, indicating that processing averted gaze generally demanded more cognitive resources during emotion recognition. This suggests that pupil dilation may reflect broader task demands or stimulus ambiguity. Specifically, masked faces showing fear triggered significant pupil enlargement, consistent with the idea that covering crucial facial cues (e.g., the mouth) increases the cognitive workload of recognizing ambiguous or uncertain expressions (Beatty, 1982; Lavín et al., 2014). This aligns with the results of Boccaccio et al. (2023), that covering the face particularly compromises the identification of emotions, including fear, sadness, and disgust. This concept corroborates our results, where pupil dilation increased when participants observed masked fearful faces, but not for anger or neutral faces. The significant dilation for incorrect angry trials, despite anger’s overall accuracy advantage, further underscores that errors in recognizing this socially salient emotion involve substantial cognitive effort. This supports the idea that fear processing, especially under face masks, is more effortful due to occlusion of diagnostic regions like the mouth, as reflected in increased pupil dilation, while even highly recognizable emotions like anger impose significant load when recognition fails.

Our pupillometry results showed that averted gaze, overall leading to greater pupil dilation than direct gaze, suggests that beyond introducing ambiguity and requiring more cognitive effort, averted gaze in emotional contexts may violate social expectations. For instance, people typically expect angry faces to look directly at them and fearful faces to look away. When this expected pattern is disrupted, the brain may engage additional resources to resolve the inconsistency, reflected in increased pupil size.

Interestingly, we found no main effect of emotion or face mask alone on pupil size. This indicates that neither emotional expression nor masking by itself was enough to significantly influence pupil responses. Instead, our findings point to the importance of the interaction between emotion, gaze direction, and masking. These findings highlight the importance of social signal ambiguity in modulating physiological responses. Pupil dilation appears to respond more strongly to dynamic combinations of cues, such as a fearful face with a mask and averted gaze, than to any one cue in isolation. Our findings point to compensatory processing mechanisms, when parts of the face were obscured by masks, participants appeared to rely more heavily on gaze direction and visible emotional cues.

Altogether, we argue that pupil dilation reflects the additional cognitive load experienced when facial cues are occluded (e.g., due to face masks) or potentially incongruent with the emotion (e.g., averted gaze for anger, direct gaze for fear). Specifically, the enlarged pupil size observed for fearful faces with a face mask likely reflects heightened ambiguity in decoding fear without mouth cues. Meanwhile, neutral expressions can also trigger pupillary changes under mismatched gaze conditions, suggesting that incongruence between an expected approach signal (anger or clarity in neutral) and an averted gaze can elevate cognitive effort (see Laeng et al., 2010; Lavín et al., 2014). Supporting this, our z-normalized analysis showed significantly greater pupil dilation in incorrect trials across all emotional categories, with the largest effect observed for angry expressions. This suggests that anger, despite being more accurately recognized behaviorally, still imposes considerable processing load when recognition fails, likely due to its heightened social salience. Thus, pupil responses may reflect not only ambiguity or perceptual difficulty, but also the motivational relevance of the emotional signal.

Our study has several limitations that must be addressed in future research. Future research should investigate how various visual barriers, such as VR headsets and medical devices, uniquely affect emotion recognition and social communication to offering insights for designing more inclusive environments. These studies would have broad implications for fields such as human-computer interaction (HCI), telecommunication, and assistive technologies. Understanding how visual barriers impact emotion recognition can inform the design of adaptive systems. Previous research has shown that pupillometry can be used to measure cognitive load in VR environments (Lee et al., 2023). HCI interfaces could utilize real-time pupil monitoring to detect cognitive load and tailor interactions accordingly. Nevertheless, this study only included three emotions (fear, anger, and neutral) chosen for their reliance on the eye region. Future research should broaden the range of emotions and intensities, and investigate which cognitive processes are most relevant in more complex decision-making. Moreover, the use of static images limits generalizability to real-world dynamic interactions where expressions and gaze naturally evolve; future work should explore how dynamic facial expressions (Cunningham and Wallraven, 2009) affect the observed patterns of accuracy, reaction time, and pupil dilation. While the present study treated masks as a form of visual occlusion that hinders access to lower-face cues, we acknowledge that real-world face masks may also carry symbolic meaning, such as signaling disease prevention or prosocial behavior. These sociocultural interpretations could influence perception and attention beyond mere occlusion effects, as previous studies have shown that face masks are not only visual barriers but also carry symbolic meanings such as health concern, trust, and social conformity (Marini et al., 2021). Future studies could examine these factors by comparing traditional face masks with non-mask occluders (e.g., black bars or blurred regions) or by assessing participants’ attitudes toward mask-wearing.

It is also important to acknowledge that although we normalized luminance across stimuli using the SHINE toolbox (Willenbockel et al., 2010), the spatial distribution of bright regions, such as the white area of surgical masks, may have varied across conditions. Particularly in the averted gaze with mask condition, the visible surface area of the white mask may have been more prominent. Given evidence that pupil size can be influenced not only by bottom-up brightness but also by the content of visual working memory and attention (Zokaei et al., 2019; Blom et al., 2016), the increased dilation observed for masked fearful faces may partially reflect these low-level visual properties. Finally, while pupil size can be modulated by perceived attractiveness (Liao et al., 2020), we consider this an unlikely confound in the present study. The strongest pupil responses were observed for angry faces, which are generally rated as less attractive or even aversive (Said et al., 2009; Todorov and Engell, 2008). Future studies may benefit from more fine-grained control of local luminance distributions across stimuli or by using dynamic normalization methods that account for white area ratios. Importantly, although we considered the possibility that gaze allocation across facial regions (e.g., eye vs. mouth) might have contributed to the observed pupil dilation effects, our supplementary AOI analysis did not support this explanation. The lack of significant effects involving gaze direction in fixation patterns suggests that the pupillary differences are more likely to reflect cognitive-emotional processing rather than gaze-induced luminance shifts. We also acknowledge that oculomotor behavior, such as saccade amplitude, may influence pupil size. Larger saccades have been linked to greater pupil dilation, potentially reflecting increased attentional effort (Wierda et al., 2012). Future studies using high-speed systems could examine how gaze shifts across facial regions contribute to pupillary responses during emotion recognition.

## Data Availability

The original contributions presented in the study are included in the article/Supplementary material, further inquiries can be directed to the corresponding author.

## Footnotes

^1^ https://osf.io/f5wtv/
