# Supplementary material for: Pupillary responses to masked and gaze-averted faces
Source: Front Psychol. 2025 Aug 12;16:1586186. doi: 10.3389/fpsyg.2025.1586186 (PMC12378493; doi:10.3389/fpsyg.2025.1586186)
Supplement: Supplementary file 1 [file Supplementary_file_1.docx]

## **Supplementary Materials**

**Supplementary Table S1.**

*Combined Repeated-Measures ANOVA Summary for Fixation Counts on Eye (AOI 1) and Mouth (AOI 2) Regions*

| **Source** | **df** | **F (AOI1: Eye)** | **p** | **η²ₚ** | **F (AOI2: Mouth)** | **p** | **η²ₚ** |
| --- | --- | --- | --- | --- | --- | --- | --- |
| Emotion | 2 | 15.29 | < .001 *** | .022 | 28.84 | < .001 *** | .041 |
| Mask | 1 | 577.48 | < .001 *** | .464 | 703.54 | < .001 *** | .513 |
| Direction | 1 | 0.19 | .662 | .000 | 0.19 | .663 | .000 |
| Emotion × Mask | 2 | 29.67 | < .001 *** | .043 | 27.03 | < .001 *** | .039 |
| Emotion × Direction | 2 | 0.86 | .423 | .001 | 2.31 | .099 | .003 |
| Mask × Direction | 1 | 0.08 | .773 | .000 | 0.11 | .738 | .000 |
| Emotion × Mask × Direction | 2 | 0.60 | .551 | .001 | 2.10 | .123 | .003 |

***Note.*** This table summarizes the repeated-measures ANOVA results for fixation counts on the eye (AOI1) and mouth/mask (AOI2) regions. Significant effects of emotion and mask were found in both regions, while gaze direction and higher-order interactions were not significant.

**Supplementary Table S2.**

*Fixation Counts on the Areas of Interest (i.e., Eye and Mouth) for Emotion and Face Mask Interaction*

| Areas Of Interest | Condition | Mean | Std. Deviation | p_bonf_ |
| --- | --- | --- | --- | --- |
| AOI 1  (Eye region) | Fear, Mask | 96.46 | 27.364 | <.001*** |
|  | Fear, No mask | 82.81 | 44.824 |  |
|  | Anger, Mask | 97.32 | 42.278 | <.001*** |
|  | Anger, No mask | 80.09 | 21.51 |  |
|  | Neutral, Mask | 96.24 | 22.616 | <.001*** |
|  | Neutral, No mask | 85.30 | 18.54 |  |
| AOI 2  (Mouth region) | Fear, Mask | .43 | 18.688 | <.001*** |
|  | Fear, No mask | 14.21 | 17.67 |  |
|  | Anger, Mask | .54 | 17.227 | <.001*** |
|  | Anger, No mask | 16.92 | 19.47 |  |
|  | Neutral, Mask | .62 | 19.845 | <.001*** |
|  | Neutral, No mask | 18.233 | 16.57 |  |

***Note.*** This table presents mean fixation counts on the eye and mouth regions across emotion and mask conditions. The results illustrate how face masks systematically reduced attention to the mouth area while preserving gaze to the eye region.

**Supplementary Figure S1.**

*Total Number of Fixations on the Areas of Interest (i.e., Eye and Mouth) for Emotion and Face Mask Interaction*


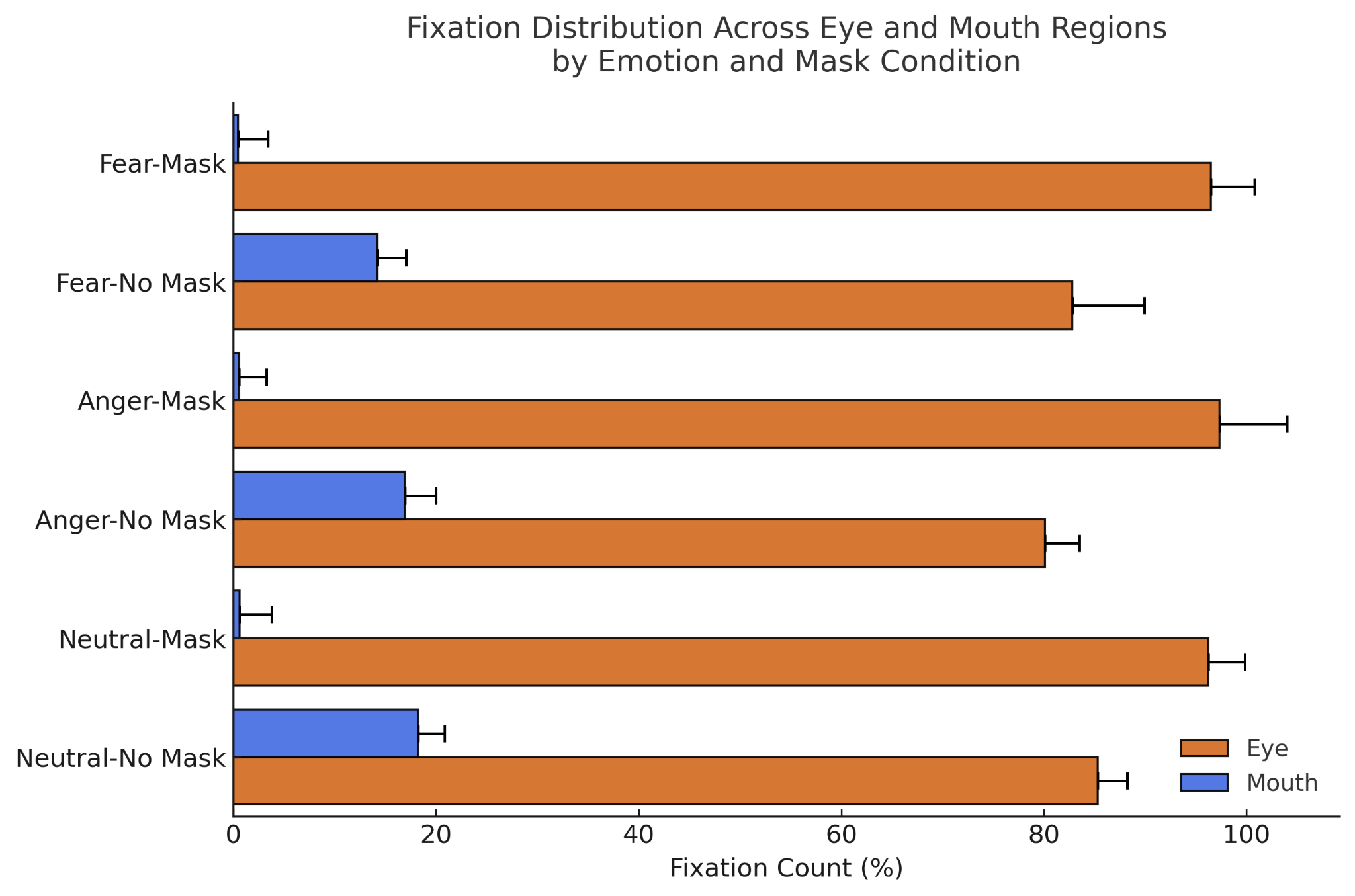


***Note.*** The figure illustrates how total fixation counts on the eye and mouth regions varied by emotion and mask condition.

**Supplementary Figure S2.**

*Confusion Matrix of Emotion Recognition Responses (% and Count)*

**
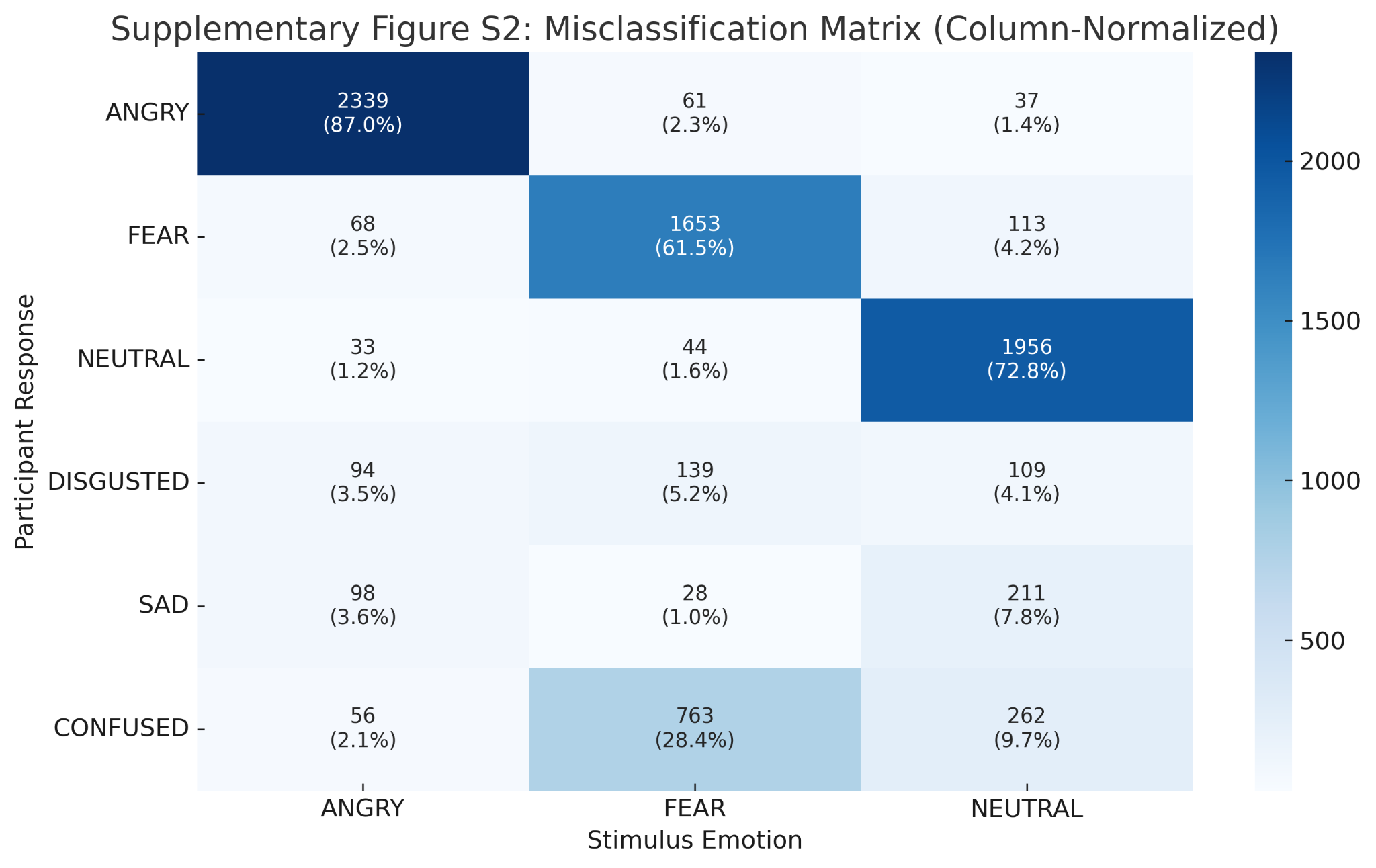
**

***Note****:* Stimuli included only Angry, Fear, and Neutral expressions, but participants responded from a six-label set. Percentages indicate the proportion of responses per row.

**Supplementary Figure S3.**

*Reaction time (in milliseconds) During the Emotion Recognition Task.*

*
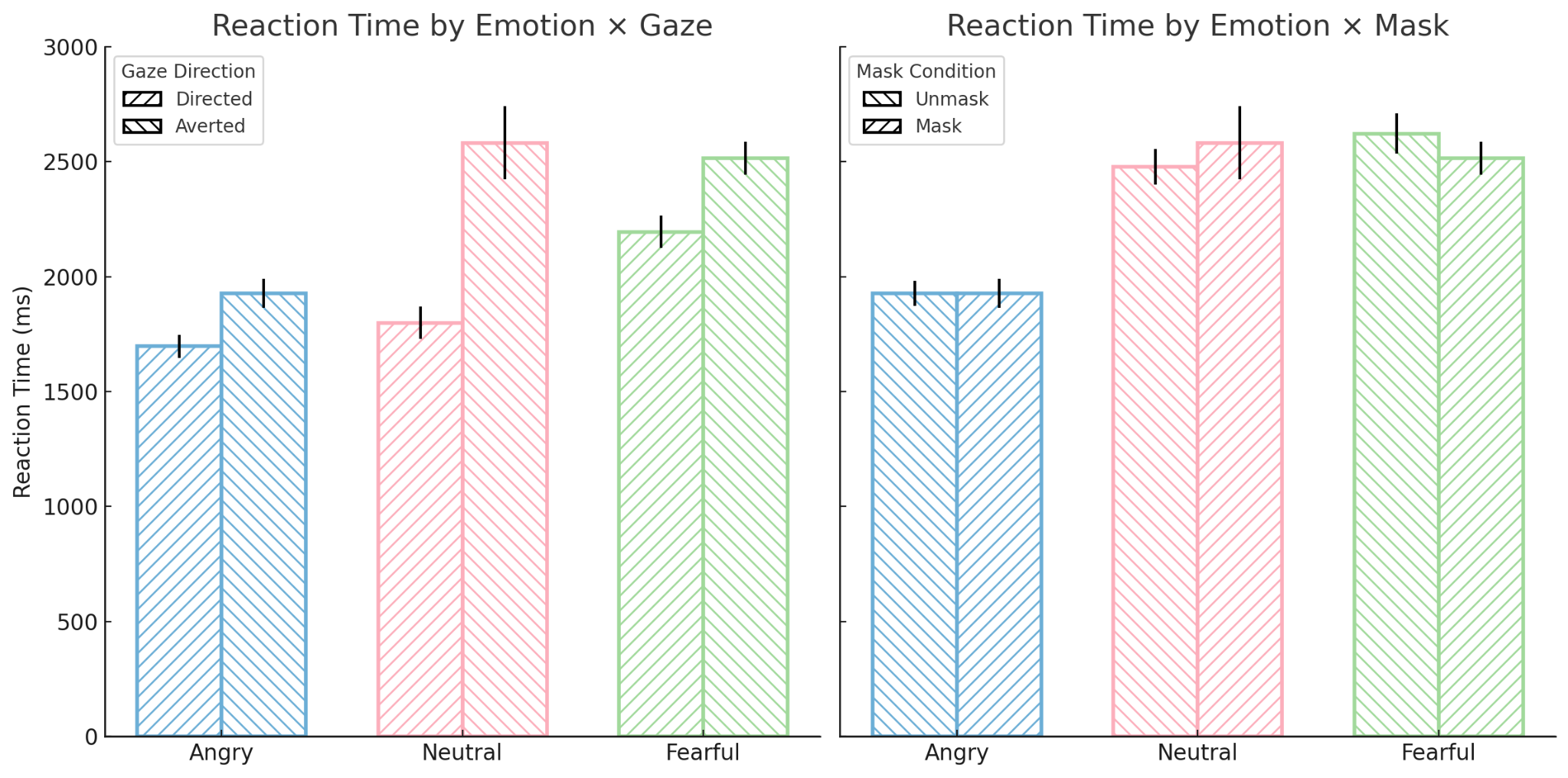
*

*Note.* Error bars represent standard errors of the mean (SE).

**Supplementary Figure S4.**

*Three-Way Interaction of Emotion, Mask, and Gaze Direction on Pupil Size*

**
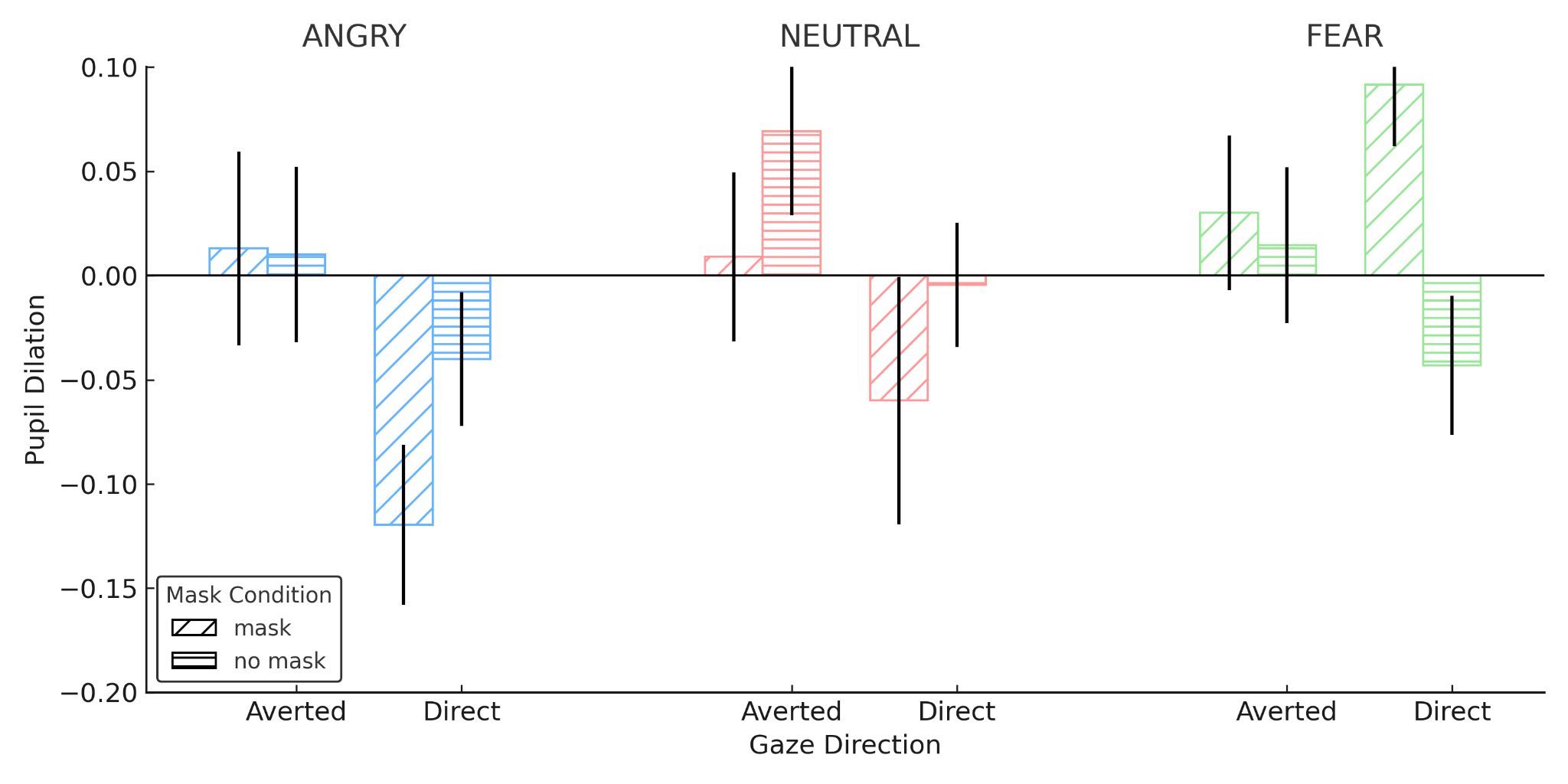
**

***Note.*** The figure displays mean pupil size as a function of emotion (fear, anger, neutral), mask condition (mask vs. no mask), and gaze direction (direct vs. averted).
